# Resonance frequency analysis-reliability in third generation instruments: Osstell mentor®

**DOI:** 10.4317/medoral.17861

**Published:** 2012-02-09

**Authors:** Mariano Herrero-Climent, Matteo Albertini, Jose V. Rios-Santos, Pedro Lázaro-Calvo, Ana Fernández-Palacín, Pedro Bullon

**Affiliations:** 1 Lecturer at the Master’s Degree in Periodontology and Implants at the University of Seville; 2 Full-time lecturer at the Department of Dentistry at the University of Seville; 3 Full-time lecturer at the Department of Preventive Medicine and Public Health at the University of Seville; 4 Full Professor at the Department of Dentistry at the University of Seville

## Abstract

Few studies assess repeatability and reproducibility in registers of resonance frequency analysis (a value of dental implant stability). Objective: Few studies assess repeatability and reproducibility in resonance frequency analyses (implant stability evaluation). This study is aimed at assessing reliability (repeatabilty and reproducibility) in the Osstell Mentor® system using the intraclass correlation coefficient (ICC) as the statistical method. Study Design: ISQ measurements of RFA were carried out by means of the Osstell Mentor® instrument in 58 implants in 19 patients. Six measurements were performed on each implant by means of two different Smart-Pegs (I and II). Three consecutive measurements were registered with each transducer. Results: Average ISQ varied from 72.43 to 72.60 and 73.26 in the first, second and third measurements, respectively with the SamrtPeg I and from 72.98 to 73.26 and 73.74 in the first, second and third measurements, respectively with the SamrtPeg II . Exactly equal values were observed in 10.43 and 12.1% of the cases with Smart-Pegs I and II, respectively. The intraclass correlation coefficient was 0.96 and 0.96 for Smart Pegs I and II, respectively. Repeatability and reproducibility was 0.97 for both Smart-Pegs I and II. Conclusions: The RFA system contributed by Osstell Mentor® renders almost perfect reproducibility and repeatability, as proven by statistical analysis carried out by means of ICC with 95% confidence level. This instrument contributes highly reliable RFA measurements in dental implants.

** Key words:**Dental implants, RFA, ISQ, implant stability, Osstell.

## Introduction

The rehabilitation of partially or completely toothless patients by means of implant supported prostheses is a predictable treatment (1).

To achieve osseointegration of dental implants, certain biological and biomechanical requirements must be met. One of the most important requirements is the absence of micro-movements during the stage of osseous cicatrization (2). In classic implant products, implants receive no functional load until bone and implant surface are closely jointed together, as this assures permanent implant stability throughout the stages that follow implant placement.

Nowadays the development of new osseophilic surfaces allows shortening loading time in implants, thus accelerating the process of bone apposition around implants (3-4). In procedures of immediate load, where the prosthesis is directly connected to the implant within seven days after the surgical stage (5), attachment primary stability (absence of clinically appreciable movements after implant insertion into the periodontium) is one of the most favoring factors of osseointegration (2).

Implant stability can be defined as the absence of clinical mobility under a specific load, which depends on the contact between implant surface and the bone surrounding the implant.

We must differentiate between primary and secondary stability. The former is determined by the pressure exerted by the implant when inserted into the carved periodontium in a calcified tissue such as a bone. The latter is the one that the implant acquires when the bone forms in direct contact with the implant surface and is determined by the process of osseointegration itself.

In this paradigm, the assessment of implant stability becomes very important to obtain successful and predictable bone-implant attachment.

Several methods have been proposed so far to assess implant stability, such as the insertion torque, the sound upon percussion, the anti-rotational torque, the response to percussion (Perio-Test®) and resonance frequency analysis (RFA).

RFA is a test to assess implant stability by measuring the frequency of implant oscillation inside the bone (6-7). A transducer connected to the implant is excited by means of an electric or magnetic impulse (depending on the type of transducer used). Thus, the implant is subjected to slight lateral force that causes lateral displacement due to elastic deformation of the bone. The frequency of the registered oscillation depends on the stiffness of bone-implant attachment: the stiffer the system is, the higher the transducer’s oscillation frequency will be. While most tests render subjective results, RFA allows objective, noninvasive assessment of implant stability (8).

There are several generations of transducers and assessment instruments. First generation transducers were constituted by an L-shaped metallic accessory made of surgical stainless steel or titanium that was coupled to (screwed on) the implant or the pillar. This accessory had two ceramic pieces at the ends: the first was excited through a sinusoidal signal of variable frequency that caused the implant to vibrate. On the other hand, the latter ceramic piece measured the response to vibration and the signal was amplified prior to comparison with the original signal by means of a frequency analyzer (9). To visualize changes in the signal, an oscilloscope and a computer were necessary.

Third generation instruments (Osstell®; Osstell AB, Gothenburg, Sweden) need no computer to complete analysis, are light, small, quick and easy to use in everyday clinic activity. Unlike previous generations, the transducer demands no calibration in 3G instruments.

Stability values are expressed in ISQ (Implant Stability Quotient) units, which range from 1 (low stability) to 100 (high stability). There is a specific transducer for each type of implant and the obtained values do not depend on the type of transducer (9).

In the first instrument to enter the market (Osstell®; Osstell AB, Gothenburg, Sweden), the transducer was connected to the instrument by means of a cable, while in the last two models (Osstell Mentor® and Osstell ISQ®, Osstell AB, Gothenburg, Sweden) the transducer known as Smart-Peg —is screwed on the implant and communicates with the instruments through electromagnetic waves.

To compare different measurements taken with the same instrument, it must be capable of reproducing highly similar values in different takes (i.e., the instrument is required to render a high degree of repeatability). Besides, the values registered by means of different Smart-Pegs must also be similar (i.e., the instrument is required a high degree of reproducibility).

Few studies in literature either assess repeatability and reproducibility in registers taken with each of the available systems or compare obtained measures with those rendered by different generations of RFA instruments.

This work is aimed at assessing the reliability (repeatability and reproducibility) of the Osstell Mentor® (Ostell AB, Gothenburg, Sweden) system.

## Material and Methods

Population of study

Measurements were taken in a sample of 58 implants placed in 19 patients among those who came to the Clinic of the Master’s Degree in Periodontology and Implants at the Faculty of Dentistry of Seville. Registers comprise from September 2008 to June 2009.

Sample size 

In a previous pilot study, the minimum size of the sample (no = 27) was determined for statistical significance p = 0.05 and confidence interval was set at 95% by means of software package N-Query Advisor 6.0.

Sample size was determined by means Bonett’s formula (10) and data analysis was carried out with software package SPSS 17.0 for MS Windows (SPSS, Chicago, USA).

Inclusion criteria

Patient inclusion criteria were:

• Patients who came to the university clinic of the Master’s Degree in Periodontology and Implants at the Faculty of Dentistry of Seville

• Over 18-year-old patients

• Collaborative patients

• Patients with Klockner Essential Cone® implants

• Patients who had completed a control or postsurgical visit

Implant inclusion criteria:

• Klockner Essential Cone® implants (SOADCO, Escaldes-Engordany, Andorra) of diameters 3.5, 4.0 or 4.5 mm, all of them with 4.5-mm platforms, and either 8-, 10- or 12-mm length

• Implants presenting no painful symptoms

• Implants presenting no clinical mobility

Implants were placed in a population of partially to completely toothless patients who were rehabilitated by means of Klockner Essential Cone® dental implants of rough surface obtained by means of sand blasting subtraction (Shot Blasted®). Measurements were taken in 58 implants, whose diameter was either 3.5, 4.0 or 4.5 mm, their platform was 4.5-mm long, and their length was either 8, 10 or 12 mm.

All implants were placed in the Master’s Degree in Periodontology and Implants at the Faculty of Dentistry of Seville during the described time period. Surgical procedures were completed by an experimented surgeon with over 10 years of surgical experience and expert in Klockner® implants and their features.

Resonance Frequency Analysis (RFA)

ISQ measurements of RFA were completed by means of the Osstell Mentor® instrument (Osstell AB, Gothenburg, Sweden).

Six measurements were completed for each implant with two different Smart-Pegs, while three consecutive measurements were registered with each transducer. These measurements were carried out by one only highly experienced dentist in the use of the Osstell Mentor® system for RFA assessment. The measurements were taken in the 58 implants consecutively regardless of the place and time of registration.

The catheter was placed at an approx. distance of 2 mm from the Smart-Peg, at an angle of 90º relative to the implant’s major axis. In all cases the catheter was vestibular- or buccal-oriented. The stage in which assessment was completed was not recorded, as the aim of our study is assessing reliability of the measurement system regardless of the circumstances that provide actual implant stability levels.

Smart-Pegs

The Smart-Pegs used were screwed directly on the implant without the interposition of the prosthesis pillar. The manufacturer’s guidelines were followed for Smart-Peg placement:

• Interposition of no soft tissue

• Transducer tightening at 5-8 Ncm manually by means of a specific plastic screwdriver

• None of the transducer’s parts is in contact with neighboring teeth

• Two new Smart-Pegs were used in each implant

• After the completion of each measurement, the transducer was completely removed from the implant. Thus, it was completely inserted and tightened for every subsequent measurement.

Statistical analysis 

To study consistency among the different consecutive measurements provided by the same instrument on the same patients, the intraclass correlation coefficients (ICC) were calculated according to the model of analysis of variance with repeated or intrasubject measurements. Together with ICCs, their intervals at 95% confidence were determined and the hypothesis that coefficients are null in the studied population was also studied. We consider that the two-factor, mixed model (11) is the suitable one to study the ICC. The values obtained with the ICC range between 0 (no consistency) and 1 (absolute consistency). There was certain consensus in the acceptance the following criterion: 0.01-0.20 SLIGHT; 0.21-0.40 AVERAGE; 0.41-0.60 MODERATE; 0.61-0.80 SUBSTANTIAL; 0.81-1.0 ALMOST PERFECT (12).

To compare the values of paired numeric variables, Student’s t test was applied for two related samples. Data analysis was completed with software package SPSS 17.0 for Windows (SPSS, Chicago, USA).

## Results

Measurements were carried out and registered in 58 implants in 19 patients. 77.6% of implants were in the posterior jaw and jawbone sections, while 56.9% were located on the jawbone and 43.1% on the jaw. 48% of the placed implants were 8-mm long, 37.9% was 10-mm long, and 13.8% was 12-mm long.

Average ISQ value in the measurements made with Smart-Peg I ranged from 72.43 (first measurement) to 72.60 (second measurement) and 73.26 (third measurement), while those taken with Smart-Peg II ranged from 72.98 (first), 73.26 (second), and 73.74 (third) ( Table 1). The average value of the three measurements completed with Smart Pegs I and II were 72.76 and 73.33, respectively.

**Table 1:**
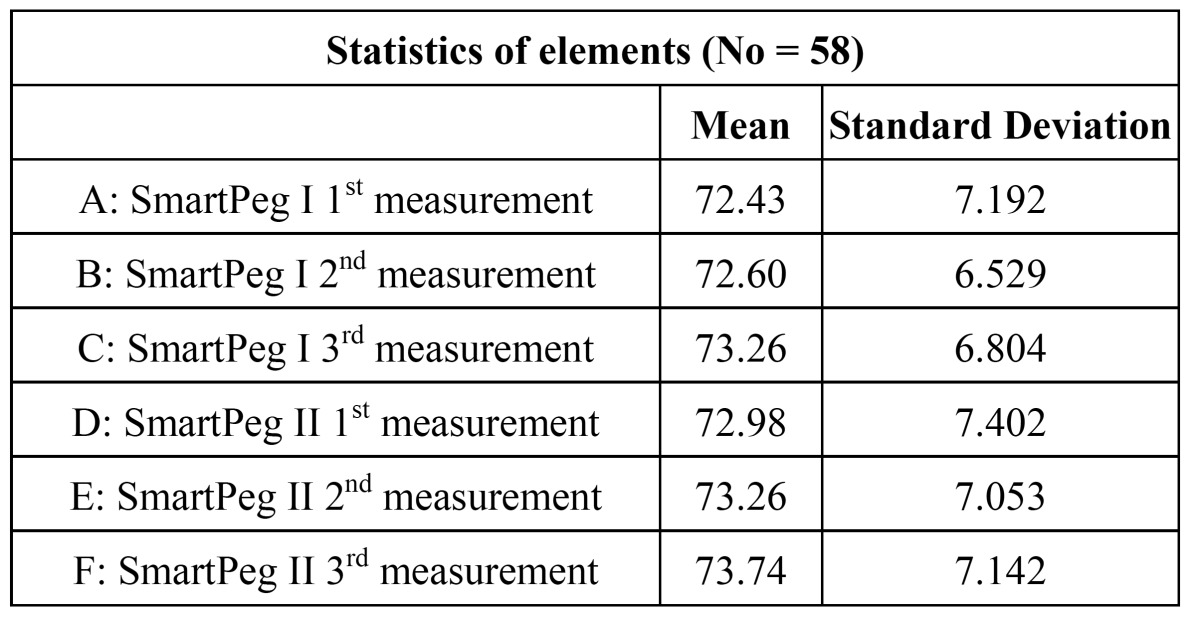
Mean obtained values expressed in ISQ units.

**Figure 1:**
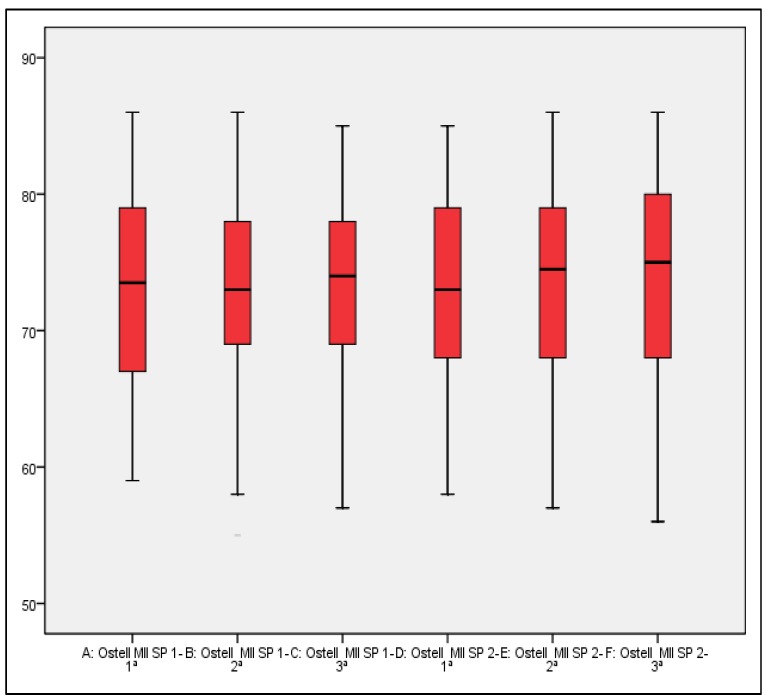
Means and interquartile difference of the obtained values with different Smart-Pegs (I and II) (3 measurements with each one).

Complete consistency was observed in the values in 10.3% and 12.1% of the cases with Smart-Pegs I and II, respectively. 74.1% and 81.0% of the registers of the first and second transducers, respectively, shows an intra-measurement difference equal to or below 3 ISQ points (  Table 2). Means and interquartile differences of the obtained values prove similar data distribution (i.e., high consistency) (Fig. 1).

**Table 2:**
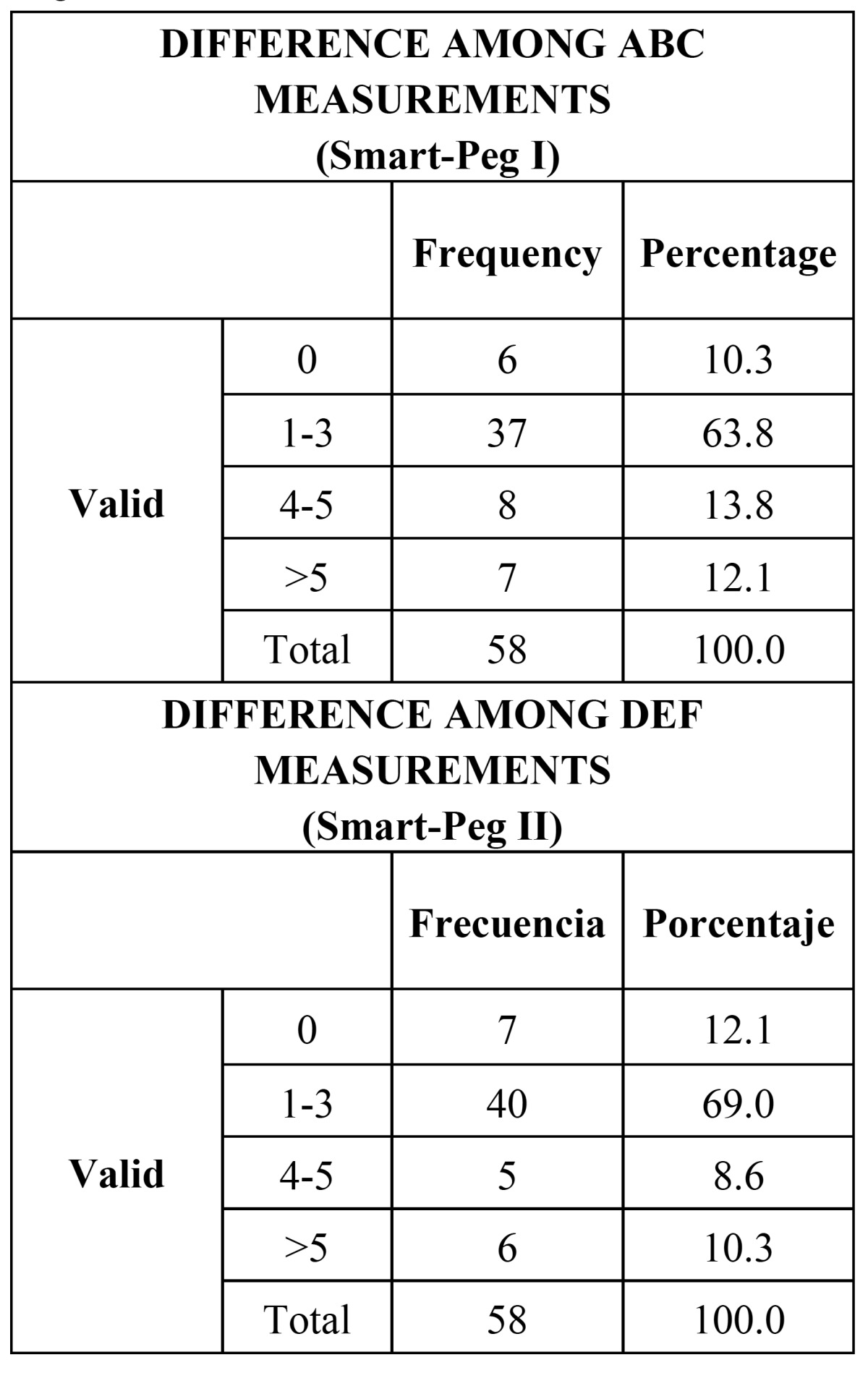
Differences among measurements with Smart-Pegs I and II.

The intraclass correlation coefficient was 0.96 for Smart Pegs I ( Table 3) and II, while the interclass correlation coefficient when both Smart-Pegs are analyzed was 0.97 ( Table 4). For both Smart-Pegs I and II repeatability was 0.96 and reproducibility was 0.97.

**Table 3:**
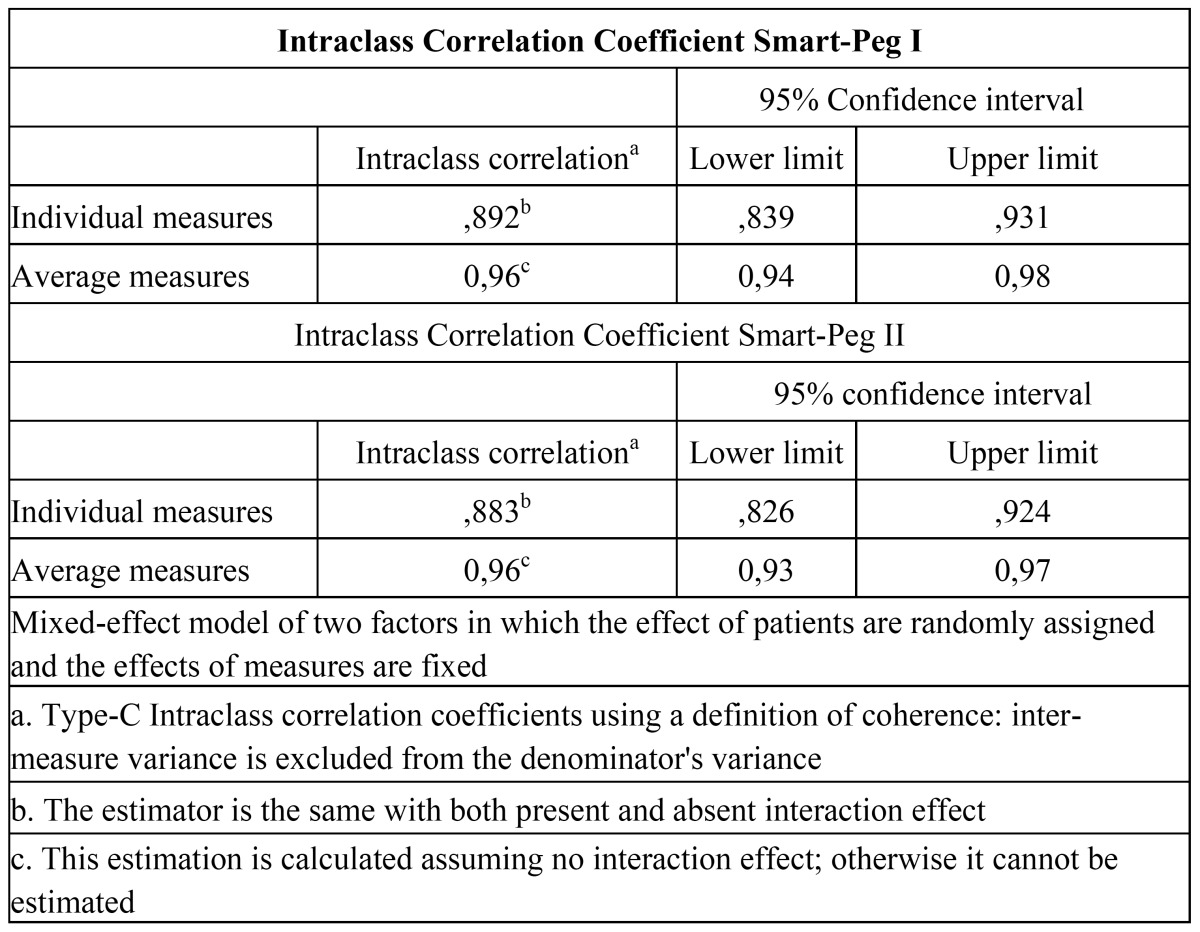
Intraclass Correlation Coefficient.

**Table 4:**
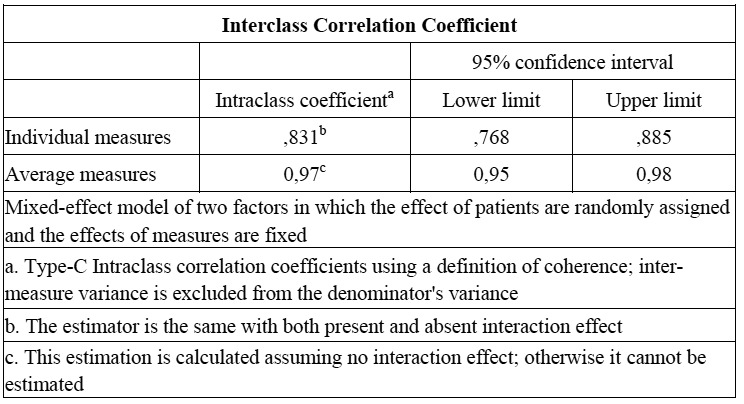
Interclass Correlation Coefficient. Smart-Pegs I and II.

## Discussion

This study is focused on the reliability of a third generation instrument aimed at measuring resonance frequency: Osstell Mentor II®.

Repeatability, defined as the assessment of the data obtained by one only transducer on one only implant, and reproducibility, defined as the assessment of the data obtained with different transducers working on one only implant at the same time, were studied. The intraclass correlation coefficient was used to measure consistency between the obtained stability values, expressed as ISQ units. These values were obtained by means of repeated measurements on 58 implants in 19 patients.

The intraclass correlation coefficient was 0.96 for Smart Pegs I and II, while the interclass correlation coefficient when both Smart-Pegs are analyzed was 0.97. The level of consistency was very high, in the “almost perfect” category.

The repeatability obtained with both Smart-Pegs I and II was 0.96; this means high consistency among repeated measurements on the same implants (i.e., the instrument is very likely to register very similar values when measuring implant stability sequentially).

Its reproducibility was 0.97. This means that there is no significant difference among the values registered with different Smart-Pegs. This finding is specially relevant at clinical level, as this instrument enables measuring stability in an implant throughout time with different transducers and comparing these measures without the need to use the same transducer.

Studies on reliability of RFA instruments are rather scarce nowadays in literature. Nedir et al. (13) conducted a clinical trial to measure stability in immediately loaded implants. These authors found that repeatability in Osstell® was 1.14% and that exact consistency was observed in 39.5% of the repeated measurements, 44.7% differed in one unit, 10.5% differed in two units, and 5.3% differed in three units. Lachmann et al. (14-15) assessed reliability in the Osstell® system in comparison with that of the Perio-Test® system. The ICC was 0.99 for the former and 0.86 for the latter. These values agree with those reported by Zix et al. (16), who completed measurements on 213 implants repeatedly with both systems. These authors concluded that both systems provide reliable implant stability measurements, although RFA is a more precise method. Valderrama et al. (17) compared the Osstell® first generation system and Osstell Mentor® second generation system. They assessed their capacity to register changes in stability levels throughout time. Similar performance was obtained with both systems, although the values registered by the latter were 10% higher than those registered by the former. Brouwser et al. (18) studied reproducibility in the RFA system in a work on desiccated jaws. These authors assessed both intra- and inter-observer measurements. Reliability ranged between average and good. Poor correlation was found between RFA and implant removal torque.

## Conclusions

The RFA system Osstell Mentor® presents almost perfect reproducibility and repeatability after statistical analysis by means of the Intraclass Correlation Coefficient (ICC) with 95% confidence.

This instrument presents high reliability to measure resonance frequency in dental implants.
